# Non-use of diabetes medication and its associated factors: a comparative analysis of female and male patients in four Sub-Saharan African countries

**DOI:** 10.1186/s12889-023-17038-z

**Published:** 2023-10-30

**Authors:** Castro Ayebeng, Joshua Okyere, Kwamena Sekyi Dickson

**Affiliations:** 1 Department of Population and Health, University of Cape Coast, Cape Coast, Ghana; 2 School of Nursing and Midwifery, College of Health Sciences, Kwame Nkrumah University of Science and Technology, Kumasi, Ghana

**Keywords:** Diabetes medication, Sex disparities, Medication non-use, Public health, Sub-saharan Africa

## Abstract

**Background:**

Globally, the burden of disease is shifting towards non-communicable diseases (NCDs), including diabetes. Sub-Saharan Africa (SSA) faces an increasing prevalence of diabetes, hindering the achievement of global health goals. This study investigates the determinants of non-use of diabetes medication, specifically exploring potential sex differences in four SSA countries.

**Methods:**

This cross-sectional study analyzed recent Demographic and Health Survey (DHS) data (2017–2021) from four SSA countries (Benin, Cameroon, Madagascar, and Mauritania). Samples included 23,695 women and 25,339 men, focusing on individuals with diabetes not using medication (248 women, 162 men). Descriptive and inferential analyses, including chi-square tests and binary logistic regression models, were conducted using Stata version 14. Odds ratios were calculated with a 95% confidence interval to determine the associations.

**Results:**

This study found that a larger proportion of female patients with diabetes (64.1%) were not using diabetes medication compared to their male counterparts (59.4%). Age influenced medication non-use in males, with older individuals exhibiting lower odds of non-usage. Higher wealth status was associated with lower odds of non-use of diabetes medications. The presence of heart disease was associated with a lower likelihood of medication non-use among females.

**Conclusions:**

This study demonstrates sex disparities, age differences, wealth status, heart disease, and country-specific variations in medication non-use. Tailored interventions for different age groups, as well as socioeconomic support, are critical, as is integrated cardiovascular and diabetes care. These actions can improve medication use and adherence, quality of life, and long-term diabetes management outcomes.

## Background

Worldwide, nations are going through an “epidemiological transition,“ with many of them now bearing a double burden of disease. In that situation, both communicable and non-communicable diseases (NCDs) are on the rise [1]. However, in recent times, NCDs have increased to become the leading cause of morbidity and death in most countries and regions. Among these NCDs, diabetes is recognised as one of the most common at the global level [2]. The 2021 International Diabetes Federation report indicates that there were 537 million people leaving with diabetes worldwide, and it is expected to increase to 784 million by 2045 [3]. The same report indicates that there were 24 million people living with diabetes in 2021 in Africa, with a projected increase of 134% by the year 2045 (i.e., translating to 55 million) [3]. Diabetes in sub-Saharan Africa (SSA) is projected to increase to 40.7 million cases by the end of 2045 [4]. Thus, making diabetes an important public health concern for SSA countries.

The statistics on the burden of diabetes in SSA is a threat to the attainment of the United Nations’ goal of reducing premature mortality from NCDs by 25% by 2025 [5], as well as a threat to achieving the sustainable development goal (SDG) target 3.4 which seeks to reduce premature mortality from NCDs by a third by 2030 [6]. This is because diabetes exacerbates the risk of developing other NCDs including chronic kidney diseases, cardiovascular diseases, and stroke [7–9].

While actions such as being physically active, healthy eating, and maintaining a healthy body weight are essential in preventing diabetes [2], the treatment and management of those with the disease are paramount. This means that diabetic medications play an important role in shaping the quality of life of persons who have been diagnosed with diabetes. The consistent and correct use of medications can avert possible complications of diabetes including both microvascular and macrovascular complications [10].

There is a plethora of research conducted across individual SSA countries that shows the magnitude of diabetes medication use [11–13]. For instance, in a study conducted in Nigeria [11], it was found that only 15.3% of persons living with diabetes accurately used medications. In Ethiopia, the proportion of non-use of medication among persons diagnosed with diabetes ranges between 34% and 63.9% [12, 13]. The non-use of diabetes medication has been found to be associated with income status, ever receiving counselling about the medication and disease, distance to the health facility, fear of complications, age, and marital status [12–14].

Despite the abundance of research conducted on non-use to diabetes medications in Sub-Saharan Africa (SSA), it remains unclear whether the established determinants vary based on the sex of individuals. Specifically, the question arises as to how the determinants of non-use of diabetes medication among males compare to those among females in SSA. Understanding the potential differences in the determinants of non-use between sexes is crucial for developing targeted interventions and strategies to improve medication adherence and health outcomes in individuals with diabetes in SSA. To address this knowledge gap and deepen the current scholarly understanding of diabetes medication use in SSA, the study examined the determinants of non-use of diabetes medication across the dimension of sex.

## Methods and materials

### Data source

The study analysed data from the most recent Demographic and Health Surveys (DHS) (2017–2021) that had the variables of interest included in the study. The DHS uses a cross-sectional study design as the conventional method. The DHS used a two-stage stratified cluster sampling method to select nationally representative samples of women in their reproductive age groups (15–49 years) and men aged 15–64 years. The DHS is suitable for our study because it gathers comprehensive information on a variety of issues, including fertility, hypertension, heart disease, diabetes, infant and child mortality, maternal (antenatal care, delivery, and postnatal care), and child (nutrition). In total, a sample of 23,695 women and 25,339 men were drawn from only 4 different SSA countries: Benin, Cameroon, Madagascar, and Mauritania which collected data on diabetes (outcome of interest). Ethical clearance for the use of the data set was not required since it was drawn from a secondary data source. However, permission for its use was secured from Measure DHS after reviewing our concept note. The dataset can be accessed at https://dhsprogram.com/methodology/survey/surveydisplay-491.cfm. We relied on the Strengthening the Reporting of Observational Studies in Epidemiology (STROBE) statement in conducting this study and writing the manuscript [15].

### Study variables and measurements

#### Outcome variable

This study centres on the “non-use of diabetes medication” as the outcome variable of interest. This was derived from the question “Are you taking medication to control your diabetes?”. The response was captured as “no = 0” and “yes = 1”.

#### Explanatory variable

Seven explanatory variables were used in agreement with empirical evidence [12–14, 16]. These variables include: Women’s age (15–19 = 1, 20–24 = 2, 25–29 = 3, 30–34 = 4, 35–39 = 5, 40–44 = 6, 45–49 = 7); and men’s age (15–19 = 1, 20–24 = 2, 25–29 = 3, 30–34 = 4, 35–39 = 5, 40–44 = 6, 45–49 = 7, 50–54 = 8, 55–59 = 9, 60–64 = 10); level of education (No education = 0, primary = 1, secondary and above = 2); wealth status (poorest = 1, poorer = 2, middle = 3, richer = 4, richest = 5); covered by health insurance (no = 0, yes = 1); diagnosed with heart disease (no = 0, yes = 1); diagnosed with hypertension (no = 0, yes = 1); and Country variable (Benin = 1, Cameroon = 2, Madagascar = 3, Mauritania = 4) (see Table 1).

**Table 1:** Background characteristics of patients with diabetes not using medication by sex

Explanatory variables	Female			Male		
	n	%	p_value	n	%	p_value
***Age*** (X^2^)	(X^2^ = 36.1519)		< 0.001	(X^2^ = 21.7466)		0.010
15–19	8	76.2		10	80.8	
20–24	32	73.7		8	59.0	
25–29	40	79.5		22	86.4	
30–34	46	77.7		12	66.0	
35–39	59	64.5		22	69.3	
40–44	42	55.8		18	75.5	
45–49	21	37.3		20	43.5	
50–54				19	48.7	
55–59				21	44.1	
60–64				10	66.4	
***Level of education***	(X^2^ = 3.3859)		0.184	(X^2^ = 1.2836)		0.526
No education	59	53.7		30	57.5	
Primary	84	69.6		35	65.0	
Secondary and above	105	67.0		97	58.1	
***Wealth index***	(X^2^ = 5.0354)		0.284	(X^2^ = 2.1229)		0.713
Poorest	22	75.4		15	70.4	
Poorer	30	52.2		10	52.3	
Middle	55	71.5		23	63.9	
Richer	54	67.7		35	66.4	
Richest	87	60.5		79	54.9	
***Subscription to health insurance***	(X^2^ = 4.3944)		0.036	(X^2^ = 4.1649)		0.041
No	225	65.7		142	62.6	
Yes	23	51.4		20	43.1	
***Diagnosed with heart disease***	(X^2^ = 10.7838)		< 0.001	(X^2^ = 1.5335)		0.216
No	236	66.4		148	61.2	
Yes	12	36.8		14	45.4	
***Diagnosed with hypertension***	(X^2^ = 0.0517)		0.820	(X^2^ = 1.5752)		0.209
No	157	62.8		103	59.8	
Yes	91	66.4		59	58.5	
**Total**	**248**	**64.1**		**162**	**59.4**	

Source: Computed from the current Demographic Health Survey (2017–2021) of four sub-Saharan African countries

#*Note*: estimates are weighted

### Analytical procedure

Descriptive and inferential analyses were carried out. The descriptive analysis involved the proportion of patients with diabetes not using medication to control their condition which was segregated by sex (male and female). It also showed the sample and the proportions of the background characteristics by the outcome variables. A chi-square test was used to examine the statistically significant difference between each explanatory variable and the outcome. Further, a binary logistic regression model was used in multivariate analysis to ascertain the significant association between the outcome variable (non-use of diabetes medication) and the respondents’ explanatory variables. Two models were fitted. In the first model, we looked at the association between the explanatory variables and the outcome variable among females. For the second model, we examine the association between the explanatory variables and the outcome variable among males. To address clustering in the hierarchical DHS data, the Huber-White technique was employed to calculate robust standard errors. This was necessary because the respondents were organized in survey clusters, which had the potential to introduce bias into the standard errors. A multicollinearity test was performed on each variable, and the results showed that the variables in the models had a mean-variance inflation factor (VIF) of 5.27 and 5.30 for the female and male data respectively, which shows the nonexistence of multicollinearity. Using a 95% confidence interval, the odds ratios for each variable were determined. The data were analysed using Stata (Version 14). All estimates were sample weighted to address any sampling bias due to under or over-sampling of participants from the total population. This was achieved by using the individual sampling weight variable, v005 and mv005 in the dataset for females and males respectively.

## Results

Table 2 summarizes the proportion of patients with diabetes by country and sex. The overall prevalence of patients with diabetes in all four countries was 1.4%, with females (1.6%) having a higher prevalence than males (1.1%). Mauritania had the highest proportion of both female (3.4%) and male (1.5%) patients with diabetes among the individual countries. Madagascar had the lowest prevalence of both female (0.6%) and male (0.6%) patients with diabetes among the sampled countries.

**Table 2:** Diabetes status by year of survey, country, and sex

Country	Survey year	Female		Male	
		**Proportion of patients with diabetes (%)**	**Non-diabetic patients**	**Proportion of patients with diabetes (%)**	**Non-diabetic patients**
Benin	2017–2018	58 (1.1)	5,266(98.9)	50(1.4)	3,615(98.6)
Cameroon	2018	127 (1.7)	7,498(98.3)	90(1.3)	6,883(98.7)
Madagascar	2021	37 (0.6)	5,837(99.4)	51(0.6)	8,980(99.4)
Mauritania	2019–2021	165 (3.4)	4,705(96.6)	83(1.5)	5,586(98.5)
**All countries**		**388 (1.6)**	**23,307(98.4)**	**274(1.1)**	**25,065(98.9)**

Source: Computed from the current Demographic Health Survey (2017–2021) of four sub-Saharan African countries

#*Note*: estimates are weighted

**Fig. 1:**
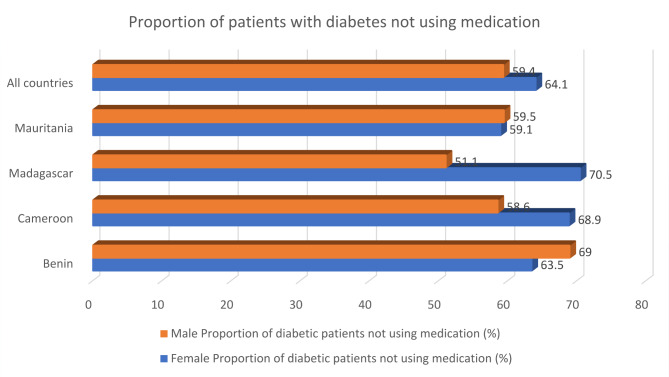
A bar graph showing the proportion of patients with diabetes not using medication by sex and country

Figure 1 shows the proportion of patients with diabetes who do not take medication. Overall, comparing females to males across all four countries, females (64.1%) outnumbered males (59.4%). The proportion of patients with diabetes who do not take medication varies by country and sex. In Benin, 63.5% of female patients with diabetes and 69.0% of male patients with diabetes were not taking medication. Compared to Cameroon, most patients with diabetes who were not taking medication were females (68.9%) and 58.6% of males. Similarly, in Madagascar, 70.5% of female patients with diabetes and 51.1% of male patients with diabetes were not taking medication. Relatedly, 59.5% of female and 59.1% of male patients with diabetes were not taking medication in Mauritania.

Table 1 presents the proportions of male and female patients with diabetes who are not using medication to control their condition. Corresponding levels of association between the independent variables and the dependent variable are also indicated based on the chi-square test. Apart from age, subscription to health insurance, and being ever diagnosed with heart disease, which showed a significant association with the likelihood of not using a medication, the other variables show no significant association with the non-use of medication. Among females, the lowest percentage of diabetes patients not using medication was in the 45–49 age group (37.3%), while the highest was in the 25–29 age group (79.5%). In contrast, among males, the lowest percentage was in the 45–49 age group (43.5%), and the highest was in the 25–29 age group (86.4%). Concerning education, there was no significant difference between the lowest and highest percentages in both sexes. The level of education did not show a significant difference between the lowest and highest percentages in both sexes. In terms of wealth, the poorest category for both females (75.4%) and males (70.4%) had the highest percentage of diabetes patients not using medication. Subscription to health insurance was significantly associated with the non-use of diabetic medication for both females and males, with the lowest percentage in the “yes” category (51.4% vs. 43.1%) and the highest percentage in the “no” category (65.7% vs. 62.6% respectively). There was a significant difference between the lowest and highest percentages of females diagnosed with heart disease, with the highest percentage in the “no” category (66.4%) and the lowest percentage in the “yes” category (36.8%). Males, on the other hand, showed no significant difference. In both sexes, there was no significant difference between the lowest and highest percentages of those diagnosed with hypertension.

### Factors associated with non-use of diabetes medication

Table 3 presents the bivariate and multivariate logistic regression analysis results on factors influencing the non-use of diabetic medication, with crude and adjusted odds ratios (OR) at 95% confidence intervals (CIs) for each explanatory variable. The results present two separate models for females and males respectively. In the adjusted model, age, wealth index, and ever-diagnosed with heart disease were inversely associated with non-use of diabetic medication among female patients with diabetes. Compared to male patients living with diabetes, age was the only significant factor influencing their non-use of medication in an inverse direction.

**Table 3:** Bivariate and multivariate logistic regression results on factors associated with non-use of diabetes medication

Explanatory variables	Female				Male			
	COR (95%CI) (Model 1)	P-value	AOR (95%CI) (Model 2)	p-values	COR (95%CI) (Model 1)	P-value	AOR (95%CI) (Model 2)	p-value
***Age***								
15–19	Ref		Ref		Ref		Ref	
20–24	1.13(0.20)	0.888	1.11(0.19,6.60)	0.910	0.63(0.12,3.47)	0.60	0.51(0.04,6.42)	0.427
25–29	1.14(0.25,8.37)	0.682	1.37(0.23,8.16)	0.730	0.81(0.15,4.32)	0.803	0.83(0.61,1.13)	0.825
30–34	1.00(0.18,5.49)	1.000	0.89(0.16,5.03)	0.899	0.58(0.12,2.78)	0.493	0.52(0.17,1.59)	0.445
35–39	0.55(0.10,2.90)	0.483	0.55(0.10,2.98)	0.486	0.60(0.14,2.56)	0.490	0.55(0.18,1.61)	0.425
40–44	0.40(0.07,2.11)	0.280	0.39(0.07,2.15)	0.278	0.65(0.14,3.12)	0.594	0.56(0.24,1.29)	0.481
45–49	0.19(0.04,1.04)	0.056	0.15(0.03,0.85)	**0.032**	0.19(0.05,0.77)	**0.020**	0.15(0.08,0.29)	**0.009**
50–54					0.23(0.06,0.94)	**0.041**	0.19(0.09,0.40)	**0.024**
55–59					0.20(0.05,0.81)	**0.023**	0.18(0.09,0.37)	**0.015**
60–64					0.20(0.04,1.01)	**0.052**	0.16(0.06,0.43)	**0.033**
***Level of education***								
No education	Ref		Ref		Ref		Ref	
Primary	1.53(0.90,2.60)	0.116	1.46(0.83,2.56)	0.248	1.55(0.70,3.41)	0.278	1.08(0.34,3.36)	0.879
Secondary and above	1.51(0.92,2.49)	0.104	1.25(0.76,2.06)	0.562	1.13(0.61,2.09)	0.703	0.97(0.31,3.08)	0.948
***Wealth index***								
Poorest	Ref		Ref				Ref	
Poorer	0.38(0.14,1.05)	0.063	0.22(0.07,0.69)	**0.013**	0.57(0.17,1.94)	0.373	0.72(0.31,1.68)	0.636
Middle	0.68(0.26,1.81)	0.445	0.42(0.13,1.35)	0.135	0.77(0.26,2.26)	0.638	1.19(0.60,2.34)	0.788
Richer	0.48(0.18,1.23)	0.127	0.29(0.08,1.02)	**0.029**	0.87(0.32,2.37)	0.786	1.19(0.56,2.54)	0.774
Richest	0.51(0.20,1.28)	0.153	0.30(0.06,1.45)	**0.042**	0.62(0.25,1.53)	0.296	0.84(0.35,2.04)	0.771
***Subscription to health insurance***								
No	Ref		Ref		Ref		Ref	
Yes	0.54(0.30,0.97)	**0.038**	0.85(0.32,2.27)	0.634	0.52(0.28,0.98)	**0.048**	0.51(0.22,1.18)	0.083
***Diagnosed with heart disease***								
No	Ref		Ref		Ref		Ref	
Yes	0.30(0.14,0.65)	**0.002**	0.23(0.16,0.33)	**0.001**	0.63(0.30,1.32)	0.218	0.68(0.29,1.61)	0.373
***Diagnosed with hypertension***								
No	Ref		Ref		Ref		Ref	
Yes	0.95(0.62,1.46)	0.820	1.46(0.85,2.52)	0.131	0.72(0.44,1.20)	0.210	0.82(0.63,1.06)	0.479
**Country**								
Benin	Ref		Ref		Ref		Ref	
Cameroon	1.43(0.74,2.76)	0.290	1.47(1.13,1.91)	0.334	0.67(0.31,1.41)	0.288	0.60(0.41,0.89)	0.248
Madagascar	1.60(0.66,3.85)	0.294	2.20(1.41,3.41)	0.123	0.78(0.33,1.83)	0.567	0.86(0.54,1.37)	0.756
Mauritania	0.81(0.44,1.50)	0.514	0.97(0.70,1.34)	0.940	0.66(0.31,1.38)	0.268	0.58(0.33,0.99)	0.246

Source: Computed from the current Demographic Health Survey (2017–2021) of four sub-Saharan African countries

COR: Crude odds ratio; AOR: Adjusted odds ratio. #Bold p-values indicate statistically significant variables associated with the outcome

Among female patients with diabetes, those aged 45–49 years had lower odds of not utilising medication to control their diabetic condition compared to young women aged 15–19 years (AOR = 0.15, 95% CI: 0.03–0.85). Similarly, among males, the likelihood of patients with diabetes not using medication declines with increasing age. Thus, those aged 45–49, 50–54, 55–59, and 60–64 years had significantly lower odds ([AOR = 0.15, 95% CI: 0.08–0.29], [AOR = 0.19, 95% CI:0.09–0.40], [AOR = 0.18, 95% CI: 0.09–0.37], and [AOR = 0.16, 95% CI: 0.06-0.0.43]) respectively, of not using diabetes medication to control their condition compared to those aged 15–19 years.

Regarding wealth index, richer (AOR = 0.29, 95% CI: 0.09–0.88) and richest (AOR = 0.30, 95% CI: 0.09–0.95) females living with diabetes were less likely not to be using medication compared to their counterparts within the poorest wealth category. However, among males, there was no significant difference across the various levels of wealth status. The results show that female patients with diabetes who had ever been diagnosed with heart disease had a lower likelihood of not using medication (AOR = 0.23, 95% CI: 0.29–1.61) than those who had never been diagnosed with heart disease. No significant disparities were observed across countries involved in this study concerning country of residence.

## Discussion

This study aimed to investigate the factors contributing to the non-use of diabetes medication by individuals across the dimension of sex. Consistent use of diabetes medication plays an important role in shaping the quality of life of persons who have been diagnosed with the condition as well as crucial for averting long-term complications. Our study shows that non-use of diabetes medication was high among males (59.4%) and females (64.1%) living with diabetes. However, the proportion of females not using the medication is higher than that of the males. This finding is aligned with previous studies conducted in both developing and developed countries such as India [17, 18], and the United States [16], and partly confirms a study conducted in Ethiopia by Araya and colleagues [12], which found 63.9% of non-use of medication among persons diagnosed of diabetes. There are several possibilities that could account for these sex disparities in medication usage. One possibility is that female patients with diabetes face greater barriers in accessing healthcare or affording medication, which may contribute to a higher proportion of them not using medication to control their condition. This gap is justified by inequalities in terms of income, education, and employment in most developing countries, including sub-Saharan African countries [19].

This study revealed that age significantly influenced the non-use of diabetes medication among both female and male respondents, with older individuals exhibiting lower odds of not using medication compared to younger age groups, which is consistent with the findings of a previous study conducted in Brazil by Tavares et al. [20]. This outcome partly corroborates the findings of Nelson et al. [21], who found higher scores of barriers to diabetes medication use among younger age groups compared to older age groups. Our finding suggests that older patients are more likely to recognize the importance of medication use due to their concerns about potential complications associated with their condition. Also, compared to younger individuals, older people might have established healthcare routines over time. Additionally, healthcare providers may prioritize medication education and emphasize the importance of adherence for older patients which might have influenced their behaviour.

Wealth status played a differential role in medication non-use between female and male diabetes. Among females, a slight improvement in household wealth significantly declines the odds of not using diabetes medication. This finding is partially supported by a study conducted in the Eastern Zone of Tigrai, Northern Ethiopia, which found that women with improved monthly income [more than 500 Ethiopian Birr (ETB)] were less likely not to be using diabetes medication [12]. Another study conducted by Kirkman et al. [16] showed related results. This finding may be attributed to several factors. One plausible explanation is that females with lower socioeconomic status might have higher healthcare access barriers, including out-of-pocket costs of diabetes medication making them less motivated to use prescribed medications. In contrast, no significant wealth-related differences in the non-use of medication were observed among males.

In this present study, the presence of heart disease was found to be associated with a lower likelihood of medication non-use among female patients with diabetes. Diabetes patients have an elevated risk of cardiovascular disease [22, 23]. As a result, people who have already been diagnosed with heart disease may become more motivated to use anti-diabetic medications to avoid further complications and maintain better diabetes control [24]. In contrast, there was no statistically significant link between heart disease and medication non-use in male patients.

### Implications for policy and practices

Policymakers and healthcare providers must work to close sex gaps in diabetes medication use. Efforts should be made to improve female patients with diabetes’ access to healthcare, including affordable medication. Strategies such as targeted outreach programmes, health education campaigns, and financial assistance programmes can help women overcome barriers to medication access and affordability. Recognising the age-related differences in medication non-use, healthcare providers should design interventions that are age-specific. Interventions for younger people could focus on raising awareness about the importance of using anti-diabetic medication and addressing barriers unique to this age group. Healthcare providers should continue to emphasise the importance of medication use and aid in developing healthcare routines for older people. Policies should also aim to reduce socioeconomic disparities in medication non-use. Providing financial assistance programmes or insurance coverage for diabetes medication can help individuals with lower socioeconomic status afford and take their medications. Furthermore, given the link between heart disease and medication non-use in female patients with diabetes, policymakers should promote the integration of cardiovascular care into diabetes management programmes. This can include encouraging regular screenings for heart disease, improving access to cardiac medications, and providing comprehensive care that addresses both diabetes and cardiovascular health.

### Strengths and limitations

The study utilized a representative and sufficient sample size, which increases the generalizability of the findings and enhances the study’s statistical power. Multiple factors influencing medication non-use were considered, including sex, age, wealth status, and presence of heart disease. This comprehensive approach provides a more nuanced understanding of the issue and allows for targeted interventions. Also, the study specifically focused on sex disparities in medication non-use, shedding light on an important aspect of healthcare inequality and calling attention to the unique challenges faced by female patients with diabetes. However, readers are cautioned to consider the following limitations of the study in interpreting the findings: first, the study employed a cross-sectional design, which limits the ability to establish causal relationships between the factors examined and medication non-use. Second, the study relied on self-reported data, which may be subject to recall bias or social desirability bias. Patients may overestimate or underestimate their medication usage, leading to potential inaccuracies in the findings. Also, the study focused on specific countries (Cameroon, Madagascar, Benin, and Mauritania), which may limit the generalizability of the findings to other regions or countries with different healthcare systems or cultural contexts. Finally, while the study considered several factors, there may be other unmeasured variables such as health literacy, social support, or healthcare provider-patient relationships, and cultural norms that could influence medication non-use were not examined in this study due to the unavailability of information on these variables in the dataset. Also, sex was not a variable in the data but rather independent files that were analysed. Therefore, we are unable to tell whether the differences in the proportion of non-use of diabetes medication between males and females were significant or not.

## Conclusion

The findings of this study emphasise the need for targeted interventions to address the factors that contribute to medication non-use among diabetics. Sex disparities, age-related differences, wealth status, presence of heart disease, and country-specific variations were identified as influential factors. Tailored interventions for different age groups and socioeconomic support for vulnerable populations are crucial. Additionally, we recommend the integration of cardiovascular and diabetes care to address the specific challenges faced by diabetes patients in different regions.

## Data Availability

The dataset(s) supporting the conclusions of this article is(are) available in the DHS repository at: http://dhsprogram.com/data/available-datasets.cfm.

## Abbreviations

DHS: Demographic and Health Surveys
NCDs: Non-Communicable Diseases
OR: Odds Ratios
SSA: Sub-Saharan African
VIF: Variance inflation factor
